# Reliability and Validity of the German Version of the Emotional Style Questionnaire

**DOI:** 10.3389/fpsyg.2021.749585

**Published:** 2021-12-23

**Authors:** Darko Jekauc, Lea Mülberger, Susanne Weyland, Fabienne Ennigkeit, Kathrin Wunsch, Janina Krell-Roesch, Julian Fritsch

**Affiliations:** ^1^Institute of Sports and Sports Science, Karlsruhe Institute of Technology, Karlsruhe, Germany; ^2^Institute of Sports Sciences, Goethe University Frankfurt, Frankfurt, Germany

**Keywords:** emotional style, questionnaire, psychometric, German, reliability, validity

## Abstract

Until recently, emotional processes have played little role in personality psychology. Based on neuroscientific findings, Davidson and colleagues proposed a theory of emotional styles, postulating six dimensions of emotional life: outlook, resilience, social intuition, self-awareness, sensitivity to context, and attention. Recently, an English version of the Emotional Style Questionnaire (ESQ) was developed and tested for reliability and validity. The aim of the present work was to test the test–retest reliability, internal consistency, construct validity, and criterion validity of the German version of the ESQ. Two separate samples consisting of 365 and 344 subjects took part in an online survey. The results of the two studies indicated satisfactory test–retest reliability and internal consistency. Regarding the construct validity, the results from Study 1 to Study 2 indicate good model fit indices. Although there was a high correlation between the subscales outlook and resilience, the analyses supported the six-factor structure postulated by Davidson and colleagues. Substantial correlations were found between the dimensions of the ESQ and other validated scales, confirming the criterion validity of the questionnaire. Our results suggest that the German version of the ESQ is a reliable and valid measurement of emotional styles. It is a feasible and economical questionnaire that can be applied in various psychology disciplines, such as personality psychology, clinical psychology, industrial psychology or sport and exercise psychology.

## Introduction

Emotions essentially make up our lives and mark distinctive life events. They influence our actions (Slovic et al., 2007) and the way we perceive others and ourselves (Forgas and Fiedler, 1996). Additionally, a repetitive experience of maladaptive emotions is related to mental diseases such as anxiety or depression (Kret and Ploeger, 2015). Usually, emotions are considered as dynamic and relatively brief psychological states that represent a reaction to relevant stimuli in a dynamic environment (Lazarus, 1991). However, emotional processes can also occur as enduring behavioral dispositions, thus indicating a personality trait.

In personality psychology, emotional processes play a rather subordinate role. For example, in the currently dominating Big Five model, only the dimension of neuroticism explicitly refers to emotional processes, whereby this personality trait can be understood as emotional stability (Costa and McCrae, 1985). However, it should be noted that emotional processes are also inherent in other personality traits of the Big Five model (c.f., Davidson and Begley, 2012), even though they are not explicitly addressed. The theoretical basis of the Big Five model is derived from the lexical approach (Allport and Odbert, 1936; Cattell, 1943), in which it is assumed that all personality traits are colloquially represented by trait words of the respective language. In contrast, Davidson and colleagues chose a different way to ground their theory of emotional styles on neuroscientific findings regarding the structure and functions of the brain (Davidson, 1993; Wheeler et al., 1993). In this sense, an emotional styles theory can be understood as a personality theory. Specifically, it is argued that “that each individual personality and temperament reflects a different combination of the six dimensions of Emotional Style” (Davidson and Begley, 2012, p. xiv). For example, for each trait of the Big Five, they provide guidance on how these constructs can be replaced by a combination of the different dimensions of emotional styles. In this way, Davidson and Begley (2012) emotional styles theory implies that the existing personality constructs of other personality theories may be redundant, not only with respect to the Big Five constructs but also other personality constructs, such as impulsivity, optimism, trait anxiety etc. (cf., Davidson and Begley, 2012, p. xiv-xv).

In the emotional styles theory, six dimensions of emotional life are postulated: outlook, resilience, social intuition, self-awareness, sensitivity to context, and attention. The emotional styles, which are based on an individual combination of these six dimensions, represent the way an individual adopts and responds to the world, and predicts the probability to feel certain emotions or moods. In several empirical studies, Davidson and colleagues have show that the proposed emotional styles were associated with specific neural networks and that indicators of each of the six dimensions can be detected in one or more brain structures (Davidson and Begley, 2012). In the following sections, the proposed six dimensions of emotional life are outlined in more detail.

Individuals scoring high on the *outlook* dimension tend to maintain positive emotions (e.g., joy, pride) in occurring diverse life situations. In the long term, this characteristic leads to a generally positive and optimistic outlook and attitude on life (Davidson and Begley, 2012). Individuals scoring low on this dimension have a tendency to experience rather short-lived positive emotions. The lack of the ability to sustain positive emotions leads to a rather pessimistic outlook in the long run. In comparison to non-depressed participants, Heller et al. (2009) found that depressed individuals fail to sustain the activity in the nucleus accumbens over time–a structure in the brain which is supposed to play an important role in the processing of positive affect and rewarding stimuli. The results of this study suggest that the ability to sustain nucleus accumbens activity over time is associated with outlook. Furthermore, high levels on the outlook dimension appear to have positive implications for health related behaviors (Pressman et al., 2019), and medication adherence (Millstein et al., 2016).

The second dimension of emotional styles theory is *resilience*, which can be described as the time it takes an individual to recover from negative emotions. A highly resilient person recovers quickly from negative emotions, such as fear or sadness. In contrast, someone with a low level of resilience recovers from negative emotions more slowly and struggles with them for a longer time (Davidson and Begley, 2012). A high level of resilience is associated with a strong neuronal connection between the amygdala and the prefrontal cortex (Kim and Whalen, 2009; Davidson and McEwen, 2012). Furthermore, research suggests that low levels of resilience increase the risk of developing a depression later in life (Laird et al., 2019).

The third dimension of the emotional styles theory is *social intuition*, describing the ability to perceive and correctly interpret signals from the social environment (Davidson and Begley, 2012). These signals may include facial expressions, body language, or verbalizations. Individuals scoring high on the social intuition dimension feel a great deal of empathy and compassion toward other individuals and are able to respond correctly to the emotional signals they receive from their environment. Dalton et al. (2005) found that individuals with higher levels of social intuition had a higher activation of the fusiform gyrus and a low activation of the amygdala. Lower levels of social intuition are related to difficulties with interpersonal relationships (Lopes et al., 2003). Autistic people characteristically have particularly low levels of social intuition (Kennedy and Adolphs, 2012).

*Self-awareness* is the fourth dimension and represents the ability to perceive interoceptive signals from one’s own body associated with emotions and to interpret them correctly (Davidson and Begley, 2012). High levels of self-awareness are associated with a strong sensitivity toward physical and emotional signals of one’s own body. Self-awareness is related to the insula, often considered the control center of the brain that regulates the perception of emotions and bodily sensations (Davidson, 2003; Davidson and Begley, 2012). The greater the activity of the insula, the greater the awareness of internal bodily processes and sensations. In this context, Bird et al. (2010) found that a lower insula activity increased the degree of alexithymia as the inability to find words about one’s own emotional experience. With regard to mental health, Ardelt and Grunwald (2018) reported that high levels of self-awareness can support healing from trauma and psychosomatic wounds.

*Sensitivity to context* is the fifth dimension and relates to the extent to which our emotional and behavioral responses are in line with the social context (Davidson and Begley, 2012). This dimension can be regarded as the outward directed version of the dimension self-awareness. In the brain, the hippocampus appears to play an important role in context perception (Davidson and Begley, 2012). Individuals scoring high on the sensitivity dimension are able to adapt their reactions and behavior to different social environments and are aware of socially valid rules, whereas individuals with a low score in this dimension are more likely todisplay socially inappropriate behavior. Ferri et al. (2013) found that individuals with higher social awareness skills showed more effective self-judgment skills in social interactions. In the context of sports, it was shown that coaches sensitive to context are more successful in adopting their behavior to the requirements of a situation (Strauch et al., 2018, 2019).

The final dimension in the emotional styles theory is *attention*. The prefrontal cortex appears to play an important role in controlling attention (Davidson and Begley, 2012). In psychology, attention is typically considered a cognitive ability (e.g., Engle and Kane, 2004). However, due to its close connection to brain regions associated with processing emotions, Davidson and Begley (2012) consider attention as a separate dimension of emotional styles. Emotional aspects of a situation require a significant portion of our attention, and the ability to filter out emotional distractions is a central skill in an individual’s emotional life. As such, individuals with higher levels of attention are able to effectively process emotional stimuli and focus on a given task. In contrast, a person with a low level of attention has more difficulties maintaining concentration and focus on the tasks at hand. Maladaptive emotion-attention interactions often result in affective disorders (Knee et al., 1987). At the same time, however, emotion-attention interactions can be improved through training and interventions (Dolcos et al., 2020).

Kesebir et al. (2019) developed the Emotional Style Questionnaire (ESQ) to assess the six dimensions of emotional styles and an integrative measure of healthy emotionality in English-speaking individuals, and tested its psychometric properties. The construct validity of the questionnaire was largely confirmed by indicating the hypothesized six-dimensional structure of the questionnaire in both exploratory and confirmatory factor analyses. In addition, regarding criterion validity, the results showed theory-consistent significant and substantial correlations between validated questionnaires and the corresponding subscales of emotional style theory. Finally, the authors also examined the 4-week test–retest reliability for the total score (*r*_tt_ = 0.89) and for each subscale (coefficients ranging from 0.73 to 0.89). The results of this study suggest that the original version of the ESQ is reliable and valid. Up to now, the ESQ has been translated into Persian and tested for its psychometric properties, which yielded similar reliability and validity coefficients to the English version (Nazari and Griffiths, 2020). Because a German version of the ESQ is currently not available, the aims were (1) to develop a German version of the ESQ, and (2) to examine its test–retest reliability, internal consistency, construct validity as well as criterion validity. For these purposes, we conducted two studies.

## Study I

The aim of Study I was to examine internal consistency, construct validity and criterion validity of the German version of the ESQ.

### Methods Study I

#### Procedure

Participants of this study were recruited through a call *via* social media, such as Facebook and Instagram. Data were collected in an online survey *via* the SoSci Survey portal (Leiner, 2019) between June 2020 and December 2020. Before the start of the study, confirmation was obtained from the data protection officer as well as from the ethics committee of the Karlsruhe Institute of Technology.

#### Participants

The study included 204 females and 140 males between the age of 18 and 83 (*M* = 34.7 years, *SD* = 13.8). Two individuals did not indicate their gender and one individual classified oneself as diverse. There were no missing data due to item non-response because the SoSci Survey Portal prompted participants to complete the questionnaire if items were not answered. Only one person terminated the survey prematurely and this person’s data were removed from the analyses. All study participants provided informed consent prior to their study participation.

#### Measurements

To examine the criterion validity of the individual subscales and the total score (healthy emotionality) of the ESQ, the constructs listed in Table 1 were used. This procedure is similar to the procedure for validation of the original ESQ (Kesebir et al., 2019).

**TABLE 1:** Validation constructs for the Emotional Style Questionnaire (ESQ).

	Outlook	Resilience	SI	SA	SC	Attention	HE
Optimism	X						
Stress management		X					
Empathy			X				
Emotional awareness				X			
Relationship skills					X		
ADHD						X	
Neuroticism							X
Flourishing							X
Vitality							X

*SI, Social intuition; SA, Self-awareness; SC, Sensitivity to context; HE, Healthy emotionality; ADHD, Self-rating questionnaire for the diagnosis of attention-deficit/hyperactivity disorder.*

##### Emotional Style

The ESQ was originally developed by Kesebir et al. (2019) to measure the six dimensions of emotional style as well as a general healthy emotionality (total score across all six dimensions). The ESQ consists of 24 items, which have to be answered on a 7-point Likert scale ranging from 1 = *strongly disagree* to 7 = *strongly agree*. Each of the six dimensions of emotional style is represented by four items in the questionnaire. For the present study, a qualified staff member (a person with a master degree in sport and exercise psychology and native in English and German) translated the items of the ESQ from English into German. A second person (a sport science master student, also native in English and German), translated the items from German back into English without knowing the items of the original instrument. A comparison of the back-translated version of the revised ESQ with the original revealed some wording differences. These were subsequently resolved by the translators, together with the authors of this study. The German and English versions of the ESQ contain the same items and scale formatting. Subsequently, the German version of the ESQ was completed by five student assistants who were asked to rate the comprehensibility of the translation. These students were excluded from participating in the main study. They were asked in an open response format which items caused difficulties in understanding. Some students reported comprehension problems with negatively worded items. Specifically, they reported that they tended to overlook that the word “nicht” (English = not). This issue was addressed by highlighting the negative word “nicht” in bold. The final German version of the ESQ can be found in the Appendix Table 1.

##### Trait Emotional Intelligence Questionnaire

The TEIQue (Petrides, 2009) measures trait emotional intelligence and consists of 153 items that are rated on a 7-point Likert scale from 1 = *strongly disagree* to 7 = *strongly agree*. For the current study, we used the short version of the TEIQue. The scale captures 15 dimensions of emotional intelligence, although only four dimensions were considered for the current study: trait optimism (8 items), stress management (10 items), empathy (9 items), and relationship skills (9 items). We used sum scores as indicators of each subscale. The German version of the TEIQue was translated and tested for psychometric properties by Freudenthaler et al. (2008). The subscales trait optimism (*r*_tt_ = 0.86), stress management (*r*_tt_ = 0.73), empathy (*r*_tt_ = 0.72), and relationship skills (*r*_tt_ = 0.60) showed sufficient internal consistencies. Both construct validity and criterion validity could be shown for this German version of the scale (Freudenthaler et al., 2008). For the current study, the subscale trait optimism was taken as indicator for the criterion validity of the ESQ dimension outlook, the subscale stress management for the criterion validity of the ESQ dimension resilience, the subscale empathy for the criterion validity of the ESQ dimension social intuition, and the subscale relationship skills for the criterion validity of the ESQ dimension sensitivity to context.

##### Big Five Inventory

The short version of the Big Five Inventory consists of ten items, which are rated on a 5-point Likert scale from 1 = *strongly disagree* to 5 = *strongly agree* (Rammstedt et al., 2017). This questionnaire measures the five personality dimensions neuroticism, extraversion, conscientiousness, agreeableness, and openness to experience. In the present study only the subscale neuroticism was used, with the total score being the sum of the two items. The results of a large-scale reliability and validity study show sufficient psychometric properties for the BFI-10 scales and items. For test–retest reliability for neuroticism of the German version, a coefficient of 0.71 was found for an interval of 6 weeks (Rammstedt and John, 2007). The results of our study show a value for Cronbach’s alpha of 0.78. Furthermore, the results confirm the construct and criterion validity of the instrument (Rammstedt et al., 2017). In the present study, neuroticism was taken as an indicator of the criterion validity of general healthy emotionality as an integrative measure across all six dimensions of emotional styles.

##### Multidimensional Assessment of Interoceptive Awareness

This questionnaire consists of 37 items and measures eight dimensions of interoception (Mehling et al., 2018). The items consist of statements that can be rated on a 6-point scale ranging from 1 = *Never* to 6 = *Always*. In our study, only the emotional awareness subscale with five items was used. The total score of this scale consists of the summed value over all five items. The German version of the questionnaire showed similar internal consistencies to the English version, high test–retest reliability, and good convergent and discriminant validity (Mehling et al., 2018). Internal consistency for the emotional awareness dimension was α = 0.86 and the test–retest reliability was 0.77 (Bornemann et al., 2015). In the present study, this subscale was taken as an indicator of the criterion validity of the ESQ dimension self-awareness.

##### Flourishing Scale

The Flourishing Scale is a measurement tool to assess social psychological well-being, which is understood as the flourishing of personality (Diener et al., 2010). The questionnaire consists of eight items rated on a 7-point Likert scale ranging from 1 = *I strongly agree* to 7 = *I absolutely disagree*. The total score of this scale consists of the summed value over all eight items. The German version was tested and validated in an intervention study (Esch et al., 2013). The internal consistency was 0.85 and the scale correlated significantly with the SF-12 (*r* = 0.57) and sense of coherence scale (*r* = 0.69). In the present study, the Flourishing Scale was taken as an indicator of the criterion validity of general healthy emotionality as an integrative measure across all six dimensions of emotional styles.

##### Self-Rating Questionnaire for the Diagnosis of Attention-Deficit/Hyperactivity Disorder

The Self-Assessment Scale (ADHD-SR; Rösler et al., 2004) for the diagnosis of adult attention-deficit/hyperactivity disorder consists of 22 items that are rated on a 4-point Likert scale (1 = *absolutely not* to 4 = *severe/almost always occurs*). The scale consists of four dimensions: attention, hyperactivity, impulsivity, and overactivity/impulsivity. For the purposes of the current study, only the attention subscale was used, which consists of nine items. The total score of this scale consists of the summed value over all nine items. The validity of the subscale was shown, with a test–retest reliability of *r*_tt_ = 0.80 and an internal consistency of α = 0.89 (Rösler et al., 2004). In the present study, this subscale was taken as an indicator of the criterion validity of the ESQ dimension attention.

##### Subjective Vitality Scale

The Subjective Vitality Scale reflects a feeling of vitality and energy and consists of seven items (Goldbeck et al., 2019). The items are rated on a 7-point Likert scale (1 = *strongly disagree* to 7 = *strongly agree*). The total score of this scale consists of the summed value over all seven items. Construct validity could be confirmed and criterion validity with a series of validated scales (e.g., life satisfaction, somatic symptoms, basic psychological needs) could be demonstrated. Internal consistency for the scale was α = 0.87 (Goldbeck et al., 2019). In the present study, the Subjective Vitality Scale was taken as an indicator of the criterion validity of general healthy emotionality as an integrative measure across all six dimensions of emotional styles.

#### Data Analysis

Attention checks were embedded in the online questionnaires to ensure higher data quality, and the data from 25 participants in Study I who failed these checks were removed from the data sets prior to any analyses (Shamon and Berning, 2020).

##### Descriptive Statistics and Reliability

In a first step, the descriptive statistics mean (*M*), standard deviation (*SD*), and internal consistency (Cronbach’s α) were calculated.

##### Confirmatory Factor Analysis

Before performing the calculations for confirmatory factor analyses, all items of ESQ were tested for deviation from the normal distribution. For eleven items, the critical ratio was greater than 5 for skewness, and for none of the items the critical ratio was greater than 5 for kurtosis. Because of this deviation from the multivariate normal distribution, we used bootstrap procedures to adjust the *p*-values and confidence intervals. The calculation included 200 bootstrap samples with a 95% confidence level. For Bollen-Stine bootstrap, the model fit was better in all 200 bootstrap samples (*p* = 0.005). Full-information maximum likelihood estimation was carried out using AMOS 25 (Arbuckle, 2017), as it has been shown that this method can efficiently handle missing data (Jekauc et al., 2012). Overall model fit was assessed using χ^2^ statistic, with a non-significant *p*-value indicating good model fit (Barrett, 2007). Because the chi-square is highly dependent on the number of subjects in the sample (Hu and Bentler, 1999), the comparative fit index (CFI) and Tucker-Lewis index (TLI) were used to examine the relative improvement in fit by comparing the proposed model to the independence model (Bentler and Bonett, 1980). CFI and TLI values of 0.95 and higher indicate a very good model fit (Bentler and Bonett, 1980; Hu and Bentler, 1999). In addition, the root-mean-square error of approximation (RMSEA) was estimated to examine the fit of the model. RMSEA values of 0.06 and below indicate a close and good model fit. To show good model fit, zero should be included in a 90% confidence interval (Dolcos et al., 2020) around the RMSEA point estimates (Hu and Bentler, 1999). To compare the model fit of two non-nested models, the Akaike information criterion (AIC) was used. Lower AIC values indicate a better model fit.

##### Criterion Validity

To examine the criterion validity of the ESQ, we calculated Pearson correlations between the ESQ dimensions and the corresponding scales or subscales used for validation.

### Results Study 1

#### Reliability and Descriptive Statistics

Descriptive statistics and coefficients for internal consistency based on Study I are shown in Table 2 The coefficients for internal consistency of all subscales exceed the α = 0.80 mark, except for the sensitivity to context dimension. All subscales of the ESQ correlated significantly with each other except for the three correlations: resilience-social intuition, resilience-sensitivity to context, and social intuition-attention. By far the highest correlation was between the dimensions of outlook and resilience with a coefficient of 0.72, suggesting that the two subscales may represent the same construct.

**TABLE 2:** Descriptive statistics of the ESQ in Study I.

Item	1	2	3	4	5	6
Outlook (1)	–	0.72**	0.17**	0.38**	0.16**	0.45**
Resilience (2)		–	0.06	0.28**	0.06	0.51**
Social intuition (3)			–	0.34**	0.35**	0.06
Self-awareness (4)				–	0.18**	0.33**
Sensitivity to context (5)					–	0.18**
Attention (6)						–
α	0.85	0.82	0.81	0.81	0.71	0.84
*M*	4.75	4.05	5.23	5.03	4.84	4.19
*SD*	1.29	1.22	1.08	1.19	1.18	1.20

*M, mean; SD, standard deviation.*

**p < 0.01.

#### Construct Validity (Confirmatory Factor Analysis)

To examine the postulated structure of the ESQ confirmatory factor analyses were conducted using full-information maximum likelihood estimation. In addition, because of the high correlation between the two subscales outlook and resilience, we also examined a model in which the two subscales were represented by one latent factor. For that purpose, we compared the 5-factor-model and the 6-factor-model from the original article (Kesebir et al., 2019). In the 5-factor model, the items in the outlook and resilience subscales loaded on the same factor, whereas in the 6-factor model each subscale had its own factor. The fit indices for the 5-factor model and 6-factor model can be seen in Table 3. In both models, all factor loadings of the individual items were significant. The 5-factor model showed a relatively poor model fit (χ^2^ = 654.6; df = 246; *p* < 0.01; CFI = 0.88; TLI = 0.86; RMSEA = 0.069), while the 6-factor model showed a good to acceptable model fit (χ^2^ = 463.6; df = 227; *p* < 0.01; CFI = 0.93; TLI = 0.92; RMSEA = 0.052). A direct comparison of the two models showed that the 6-factor model had a lower (AIC = 637.6) and thus a better model fit than the 5-factor model (AIC = 810.8).

**TABLE 3:** Model fits of the 5-factor and 6-factor models (Study I).

Model	χ^2^	*df*	*p*	*CFI*	*TLI*	*RMSEA*	*AIC*
6-factor model	463.6	227	<0.01	0.93	0.92	0.052	637.6
5-factor model	654.6	246	<0.01	0.88	0.86	0.069	810.8

*χ^2^, Chi-square; df, degrees of freedom; CFI, comparative fit index; RMSEA, root mean square error of approximation; AIC, Akaike information criterion.*

#### Criterion-Related Validity

The results of the criterion-related validity of Study I are presented in Table 4. The shaded cells represent the indicators used for criterion validity of the individual dimensions. The results indicate that—as expected—the subscales of the ESQ correlated most strongly with their corresponding validation instruments. The subscale outlook had the strongest correlation with trait optimism (*r* = 0.78), the subscale resilience with stress management (*r* = 0.68), the subscale social intuition with empathy (*r* = 0.72), the subscale self-awareness with emotional awareness (*r* = 0.43), the subscale sensitivity to context with relationship skills (*r* = 0.42) and the subscale attention with the attention subscale of ADHD-SR (*r* = 0.66). Moreover, general healthy emotionality, based on the items of all subscales, correlated significantly with the Neuroticism (*r* = −0.45), the Flourishing Scale (*r* = 0.71) und Subjective Vitality (*r* = 0.64). In addition to these expected correlations, outlook and resilience correlated substantially with neuroticism, Flourishing Scale and Subjective Vitality Scale.

**TABLE 4:** Correlations between the ESQ and the corresponding (sub) scales used for validation (Study II).

		Trait optimism (TEIQue)	Stress management (TEIQue)	Empathy (TEIQue)	Emotional awareness (MAIA)	Relationship skills (TEIQue)	ADHD-SR	Neuroticism (BF-10)	FS	STVS
ESQ subscales	Outlook	0.78**	0.61**	0.23**	0.15**	0.27**	−0.39**	−0.60**	0.66**	0.70**
	Resilience	0.56**	0.68**	0.09	0.01	0.22**	−0.37**	−0.65**	0.47**	0.53**
	SI	0.19**	0.03	0.72**	0.32**	0.43**	–0.05	0.02	0.36**	0.20**
	SA	0.36**	0.29**	0.37**	0.43**	0.29**	−0.34**	−0.23**	0.45**	0.40**
	SC	0.21**	0.13*	0.40**	0.15*	0.42**	−0.29**	0.02	0.27**	0.10
	Attention	0.40**	0.54**	0.09	0.05	0.15*	−0.66**	−0.53**	0.43**	0.43**
	General healthy emotionality	0.68**	0.63**	0.49**	0.29**	0.47**	−0.57**	−0.54**	0.71**	0.64**

*ESQ, Emotional Style Questionnaire; SI, Social intuition; SA, Self-awareness; SC, Sensitivity to context; AT, Attention; TEIQue, Trait emotional intelligence questionnaire; MAIA, Multidimensional assessment of interoceptive awareness; ADHD-SR, Self-assessment scale for the diagnosis of adult attention-deficit/hyperactivity disorder; BF-10, Big Five Inventory (Short Form); FS, Flourishing scale; STVS, Subjective Vitality Scale.*

*p < 0.05, **p < 0.01.

### Discussion Study I

The results of Study I indicate that the German version of the ESQ has sufficiently high reliability with respect to the Cronbach’s alpha coefficients. Between most of the subscales of the questionnaire, the correlations are significant, indicating an interdependence of the constructs. The correlation between Outlook and Resilience is particularly strong, suggesting that they may be the same construct. However, this assumption could be invalidated when comparing the 5-factor model with the 6-factor model. The 6-factor model showed better model fit than the 5-factor model. Moreover, criterion-related validity of the different subscales of the questionnaire as well as the general health emotionality could be supported.

## Study II

The aim of Study II was to examine internal consistency, test–retest reliability and construct validity of the German version of ESQ.

### Methods Study II

#### Procedure

Study participants from two German universities were recruited from classes in their respective sport science programs. Between June 2020 and December 2020, data were collected in an online survey *via* the SoSci Survey portal (Leiner, 2019). At the beginning of a session (e.g., a lecture), the lecturer provided a SoSci link and asked students to click on the link and complete the questionnaire. Participants were asked if they had already completed the questionnaire in another course. If this was the case, these students were asked not to complete the questionnaire in order to prevent double participation in the study. In order to estimate test–retest reliability, participants were surveyed twice with an interval of 4 weeks between the measurements. Before the start of the study, the consent of the data protection officer as well as the ethics committee of the Karlsruhe Institute of Technology were obtained.

#### Participants

For this study, 194 females and 171 males aged between 18 and 24 years (*M* = 22.1 years, *SD* = 2.9) took part. All participants were students of the bachelor’s or master’s degree program with sports science as major and provided informed consent prior to their study participation. The sample for the retest measurement involved 110 female and 86 male participants aged between 18 and 24 years (*M* = 22.1 years, *SD* = 2.9) who had already participated in the first measurement. Again there were no missing data due to item non-response because the SoSci Survey Portal prompted participants to complete the questionnaire if items were not answered.

#### Measurement

Emotional Style was measured with the German version of the ESQ described above.

#### Data Analysis

Attention checks were embedded in the online questionnaires to ensure higher data quality, and the data from 14 participants in Study II who failed these checks were removed from the data sets prior to any analyses (Shamon and Berning, 2020).

##### Descriptive Statistics and Reliability

The descriptive statistics mean (*M*), standard deviation (*SD*), test–retest-reliability (*r*_tt_) and internal consistency (Cronbach’s α) were calculated. The test-retest reliability of the German ESQ items within a 4-week period was estimated by the Pearson product-moment correlation coefficient.

##### Confirmatory Factor Analysis

The same procedure as in Study I was performed.

### Results Study II

#### Reliability and Descriptive Statistics

Descriptive statistics and reliability estimates of Study II are provided in Table 5. The coefficients of the test–retest reliability for a period of 4 weeks for the different ESQ-subscales range from 0.64 to 0.85, with the outlook subscale having the highest retest reliability and the self-awareness subscale having the lowest. Cronbach’s α values range from α = 0.73 (self-awareness) to α = 0.80 (outlook). Some correlations between subscales were significant, with most significant correlations being moderate to small (*r* = 0.37–0.11). The highest correlation by far was again the correlation between outlook and resilience at 0.66.

**TABLE 5:** Reliability and descriptive statistics of the ESQ in Study II.

Item	1	2	3	4	5	6
Outlook (1)	–	0.66**	0.08	0.37**	0.01	0.36**
Resilience (2)		–	0.03	0.33**	0.00	0.32**
Social intuition (3)			–	0.15**	0.22**	0.02
Self-awareness (4)				–	0.11*	0.33**
Sensitivity to context (5)					–	0.20**
Attention (6)						–
Retest reliability	0.85	0.75	0.76	0.64	0.76	0.80
Cronbach’s α	0.80	0.80	0.78	0.73	0.76	0.80
*M*	4.99	4.29	5.39	5.18	5.00	3.93
*SD*	1.05	1.10	0.84	0.93	1.15	1.03

*M, mean; SD, standard deviation.*

*p < 0.05, **p < 0.01.

#### Construct Validity

To examine the postulated structure of the ESQ and to validate the results of Study I confirmatory factor analyses were conducted. Again, because of the high correlation between the two subscales outlook and resilience, we examined the model in which the two subscales were represented by one latent factor. The fit indices for the 5-factor model and 6-factor model can be seen in Table 6. In both models, all factor loadings of the individual items were significant. The 5-factor model showed a relatively poor model fit (χ^2^ = 505.5; df = 246; *p* < 0.01; CFI = 0.90; TLI = 0.87; RMSEA = 0.054), while the 6-factor model showed a good to acceptable model fit (χ^2^ = 451.1; df = 227; *p* < 0.01; CFI = 0.92; TLI = 0.89; RMSEA = 0.050). A direct comparison of the two models showed that the 6-factor model had a lower (AIC = 577.1) and thus a better model fit than the 5-factor model (AIC = 669.5).

**TABLE 6:** Model fits of the 5-factor and 6-factor models (Study II).

Model	χ^2^	*df*	*p*	*CFI*	*TLI*	*RMSEA*	*AIC*
6-factor model	451.1	227	< 0.01	0.92	0.89	0.050	577.1
5-factor model	505.5	246	< 0.01	0.90	0.87	0.054	669.5

*χ^2^, Chi-square; df, degrees of freedom; CFI, comparative fit index; RMSEA, root mean square error of approximation; AIC, Akaike information criterion.*

### Discussion Study II

The results of Study II also indicate that the German version of the ESQ has sufficiently high reliability with respect to the Cronbach’s alpha coefficients and test–retest reliability. Some subscales correlate significantly with each other. Similar to the results of Study I, the correlation between outlook and resilience is particularly strong, suggesting that they may be the same construct. Again, this assumption could not be supported when comparing the 5-factor model with the 6-factor model. The 6-factor model showed better model fit than the 5-factor model.

## General Discussion

Building on the findings of affective neuroscience, the theory of emotional styles with six different dimensions was developed (Davidson, 1993; Wheeler et al., 1993). In relation to this theory, a questionnaire was recently developed to measure these six dimensions of emotional styles, which until now could only be studied using neuroscientific methods (Kesebir et al., 2019). The present study aimed to investigate the test–retest reliability, internal consistency, construct validity, and criterion validity of the German version of the ESQ, which was developed based on the original English version of the ESQ. The results of both Study I and Study II indicate that the German version of the ESQ is a valid and reliable instrument to assess healthy emotionality.

The test–retest reliability of the German version of the ESQ was satisfactory, with the outlook dimension showing the highest values and the self-awareness dimension and the sensitivity to context dimension the lowest. Overall, however, the estimates of reliability in our study are comparable or slightly lower than the reliability estimates of the English and Persian versions of the ESQ (Kesebir et al., 2019). In both studies, the reliability coefficients of both the subscale scores as well as the total score significantly exceeded the 0.80 benchmark. One possible explanation could be that participants in our studies completed the questionnaire digitally, whereas participants in the studies by Kesebir et al. (2019) and Nazari and Griffiths (2020) had used a paper-pencil form or a mixture of paper-pencil and digital forms.

After high correlations were found between the subscales outlook and resilience, the question arose whether the two subscales are indicators of the same latent factor. To investigate this issue, the five-factor model, in which outlook and resilience load on one latent factor, was compared with the postulated six-factor model. The results of the confirmatory factor analysis based on the samples of the two studies indicate a superiority of the postulated six-factor model over the five-factor model. Kesebir et al. (2019) also found similarly high correlations between the two subscales outlook and resilience. However, the authors argued for the distinctness of the two constructs, due to different underlying neural circuits. From a theoretical point of view, however, it may be logical to assume that resilience, in the sense of recovering quickly from setbacks, is a prerequisite for a positive outlook. Individuals can only have a positive outlook in the sense of maintaining positive emotions over time if they are able to recover quickly from setbacks and adversities. However, some theorists argue (see, e.g., Southwick et al., 2014) that resilience refers not only to rapid recovery from setbacks, but also to an optimistic tendency to see improvements in the future. In this sense, outlook would be one aspect of resilience. Thus, further studies are needed to examine the difference between the two constructs at both the psychometric level as well as underlying neural levels.

In terms of criterion validity, the results show that the individual subscales of the ESQ correlate with the corresponding scales, as expected. Comparable correlations were found in the study by Kesebir et al. (2019), although we used different criteria in some cases. The optimism subscale from the Trait Emotional Intelligence Questionnaire correlated highest with the outlook subscale, the stress management subscale with the resilience subscale, the empathy subscale with the social intuition subscale, the relationship skills subscale with the sensitivity to context subscale. In contrast to the study by Kesebir et al. (2019), the emotional awareness subscale of the Multidimensional Assessment of Interoceptive Awareness (MAIA) scale had a rather low correlation with the self-awareness subscale of the ESQ. The self-assessed ADHD scale also correlated weakly to moderately with the attention subscale only. The healthy emotionality total score (ESQ) as well as outlook and resilience correlated highest with trait optimismstress management as two subscales of TEIQ, neuroticism as a subscale of Big Five, Flourishing Scale and the Subjective Vitality Scale. This could be an indicator that the two highly correlated constructs resilience and outlook are the central constructs of the healthy emotionality total score. Overall, the significant correlations confirm the criterion validity of the German version of the ESQ, although the correlations were somewhat lower than in the study by Kesebir et al. (2019).

The present study, based on two different samples, has a number of strengths and weaknesses. One strength is that we tested reliability not only in terms of internal consistency, but also in terms of test–retest reliability. Construct validity was tested by confirmatory factor analyses in separate samples. In addition, we examined criterion validity using validated and established questionnaires. A limitation of this study is that only self-report measures were used for criterion validation. Future studies could use neuroscience methodology to further establish the validity of the questionnaire. Another limitation pertains to the student sample of Study II, which is not representative of the German population. For the generalizability of the results, it would certainly have been helpful if more diversified samples could have been drawn. Thus, future research should apply the ESQ to larger studies with representative, larger samples.

There are many implementations of the ESQ, given that dealing with emotions is a central part of human interaction. In clinical psychology, the ESQ could be used for diagnostic purposes. In occupational psychology, resilience, social intuition, and context sensitivity could take an important role in activities that require soft skills and interactions with people (e.g., Landa et al., 2008). In educational psychology, attention is central for successful learning (e.g., Lauth et al., 2006). In sports psychology, resilience as the ability to deal with setbacks and attention is a central predictor for success (e.g., Gonzalez et al., 2016; Jekauc and Brand, 2017; Kopp and Jekauc, 2018). Team athletes and coaches need a considerable degree of social intuition and sensitivity to context in order to perceive or correctly interpret the social-emotional signals of teammates or opponents (e.g., Lee et al., 2018; Strauch et al., 2019).

## Conclusion

Overall, the conclusion can be drawn that the German version of the ESQ is sufficiently reliable and valid to use in research and practice. The development of the questionnaire opens up new possibilities to address a wide variety of questions that previously could only be addressed in the context of neuroscience studies. The application of the questionnaire is manifold and spans different disciplines of psychology, such as occupational psychology, educational psychology, sport psychology, or clinical psychology. Using this questionnaire, it is now possible to exploit the potentials of the emotional styles approach and to complement social science research with neuroscientific findings.

## Data Availability Statement

The raw data supporting the conclusions of this article will be made available by the authors, without undue reservation.

## Ethics Statement

The studies involving human participants were reviewed and approved by Ethics Committee of the Karlsruhe Institute of Technology. The patients/participants provided their written informed consent to participate in this study.

## Author Contributions

DJ and LM wrote the manuscript and conducted the statistical calculations. DJ and JF conceptualized the study. All authors organized the data collection, helped edit the manuscript, and approved the final version of the submitted manuscript.

## Conflict of Interest

The authors declare that the research was conducted in the absence of any commercial or financial relationships that could be construed as a potential conflict of interest.

## Publisher’s Note

All claims expressed in this article are solely those of the authors and do not necessarily represent those of their affiliated organizations, or those of the publisher, the editors and the reviewers. Any product that may be evaluated in this article, or claim that may be made by its manufacturer, is not guaranteed or endorsed by the publisher.

## Appendix

**TABLE A1:** Emotional Style Questionnaire (ESQ)–German Version.

Bitte geben Sie ihre Übereinstimmung mit den folgenden Aussagen anhand der Skalen an.							
**Emotional Style Questionnaire**	**Stimme überhaupt nicht zu**	**Stimme nicht zu**	**Stimme eher nicht zu**	**Stimme weder zu noch nicht zu**	**Stimme eher zu**	**Stimme zu**	**Stimme absolut zu**
1 Wenn mir etwas Gutes passiert, hält diese positive Stimmung **nicht** lange an.	□	□	□	□	□	□	□
2 Es fällt mir schwer, zu meiner Ruhe zurückzufinden, nachdem mir etwas Negatives passiert ist.	□	□	□	□	□	□	□
3 Wenn ich mit Menschen rede, lasse ich mich immer auf deren Gefühlszustand ein.	□	□	□	□	□	□	□
4 Es kann längere Zeiträume geben, in denen ich mir über meine eigenen körperlichen und emotionalen Zustände nicht bewusst bin.	□	□	□	□	□	□	□
5 Manchmal hat man mir gesagt, dass ich mich sozial unangemessen verhalten hätte.	□	□	□	□	□	□	□
6 Ich kann mich gut zu konzentrieren.	□	□	□	□	□	□	□
7 Ich bin sehr gut darin, die positiven Seiten in etwas zu sehen.	□	□	□	□	□	□	□
8 Wenn ich einen Rückschlag erfahre, nimmt mich das nicht lange mit.	□	□	□	□	□	□	□
9 Ich bin nicht besonders gut darin, die Emotionen anderer Menschen zu erkennen.	□	□	□	□	□	□	□
10 Normalerweise bin ich mir meiner Gefühle sehr bewusst.	□	□	□	□	□	□	□
11 Ich habe auf der Arbeit Rückschläge erlitten oder mich mit Freunden/Freundinnen gestritten, weil mein Verhalten anscheinend nicht akzeptabel war.	□	□	□	□	□	□	□
12 Auch in Situationen, in denen viel los ist, lass ich mich nicht leicht ablenken.	□	□	□	□	□	□	□
13 Mir fällt es leicht, hoffnungsvoll in die Zukunft zu blicken.	□	□	□	□	□	□	□
14 Wenn ich schlecht gelaunt bin, hält dies normalerweise lange an.	□	□	□	□	□	□	□
15 Ich habe ein gutes Gespür für die Emotionen anderer Menschen.	□	□	□	□	□	□	□
16 Ich bin **nicht** gut darin, meine eigenen Gefühle zu erkennen.	□	□	□	□	□	□	□
17 Ich habe manchmal Dinge gemacht, die andere für taktlos oder peinlich hielten.	□	□	□	□	□	□	□
18 Manchmal habe ich das Gefühl, dass ich mich **nicht** richtig konzentrieren kann, weil meine Gedanken bei anderen Dingen sind.	□	□	□	□	□	□	□
19 Wenn die Dinge schlecht laufen, fällt es mir schwer zu glauben, dass sie sich trotzdem zum Guten wenden werden.	□	□	□	□	□	□	□
20 Ich erhole mich schnell, wenn etwas **nicht** so läuft, wie ich es möchte.	□	□	□	□	□	□	□
21 Ich kann spüren, wenn eine Person etwas stört, indem ich sie nur anschaue.	□	□	□	□	□	□	□
22 Normalerweise achte ich **nicht** auf das, was in meinem Körper vor sich geht.	□	□	□	□	□	□	□
23 Wenn andere Menschen etwas für unangemessen halten, bin ich oft anderer Meinung.	□	□	□	□	□	□	□
24 Wenn ich von etwas abgelenkt werden, dauert es lange, bis ich mich wieder konzentrieren kann.	□	□	□	□	□	□	□
